# Poor sleep quality is associated with fatigue and depression in early Parkinson's disease: A longitudinal study in the PALS cohort

**DOI:** 10.3389/fneur.2022.998103

**Published:** 2022-09-01

**Authors:** Matthew Rui En Koh, Cong Yang Chua, Samuel Yong-Ern Ng, Nicole Shuang-Yu Chia, Seyed Ehsan Saffari, Regina Yu-Ying Chen, Xinyi Choi, Dede Liana Heng, Shermyn Xiumin Neo, Kay Yaw Tay, Wing Lok Au, Eng-King Tan, Louis Chew-Seng Tan, Zheyu Xu

**Affiliations:** ^1^Lee Kong Chian School of Medicine, Nanyang Technological University, Singapore, Singapore; ^2^National Neuroscience Institute, Singapore, Singapore; ^3^Centre for Quantitative Medicine, Duke-NUS Medical School, National University of Singapore, Singapore, Singapore; ^4^Duke-NUS Medical School, National University of Singapore, Singapore, Singapore

**Keywords:** Parkinson's disease, sleep, fatigue, depression, non-motor symptoms

## Abstract

**Background:**

Sleep disorders are common in Parkinson's disease (PD). However, the longitudinal relationship between sleep quality and the other non-motor symptoms of PD has not been well characterized, especially in early PD.

**Objective:**

To explore the value of baseline sleep quality in predicting the progression of other non-motor symptoms in early PD.

**Methods:**

109 early PD patients were recruited to the study. Patients were stratified into good and poor sleepers using the Pittsburgh Sleep Quality Index (PSQI). Assessments performed at baseline and 1 year follow-up included the Epworth Sleepiness Scale, Fatigue Severity Scale, Non-Motor Symptom Scale, Geriatric Depression Scale, Hospital Anxiety and Depression Scale, Apathy Scale, Montreal Cognitive Assessment and detailed neuropsychological assessments. Multivariable linear regression was performed at baseline to investigate differences in clinical scores between poor and good sleepers, while multivariable regression models were used to investigate associations between sleep quality and progression of test scores at 1 year follow-up.

**Results:**

59 poor sleepers and 50 good sleepers were identified. At baseline, poor sleepers had greater HADS anxiety scores (*p* = 0.013) [2.99 (95% CI 2.26, 3.73)] than good sleepers [1.59 (95% CI 0.75, 2.42)]. After 1 year, poor sleepers had greater fatigue (FSS scores +3.60 as compared to −2.93 in good sleepers, *p* = 0.007) and depression (GDS scores +0.42 as compared to −0.70, *p* = 0.006).

**Conclusion:**

This study shows a longitudinal association between sleep quality, fatigue, and depression in early PD patients, independent of medication effect and disease severity, this may support the hypothesis that a common serotonergic pathway is implicated in these non-motor symptoms.

## Introduction

Parkinson's Disease (PD) is a neurodegenerative disorder. Other than motor symptoms, non-motor symptoms including cognitive dysfunction, sleep disorders, affective disorders, and fatigue can occur throughout the disease course (1, 2), which contribute significantly to disease and caregiver burden (3, 4).

Sleep disorders that commonly occur in PD include Rapid Eye Movement sleep behavior disorder (RBD), excessive daytime sleepiness (EDS) and insomnia (5). Associations between sleep disorders and the other non-motor symptoms of PD have been observed: RBD, EDS and poor sleep quality have all been linked to poor cognition, particularly in the domains of attention and executive function (6–9), while poor sleep quality has been linked to depression and anxiety (10).

Fatigue is defined as “*a sense of exhaustion unexplained by drug effects, other medical, or psychiatric disorders”* (11), and is considered an independent non-motor symptom in PD (12). Fatigue can occur even in early PD, and is estimated to have a prevalence rate of around 50% across all disease stages (13). The neuropathological basis for fatigue in PD is not fully understood, with non-dopaminergic pathways implicated in its pathogenesis (14).

While several studies have examined the impact of RBD and EDS on the longitudinal progression of non-motor symptoms in PD (6, 7, 15–17), current literature on the impact of sleep quality on other non-motor symptoms is limited, especially in early PD, with most studies demonstrating only cross-sectional associations. Stavitsky et al. (18) and Kim et al. (19) showed in non-early PD cohorts that poor sleep quality was associated with poorer cognitive performance, while Junho et al. (10) and Rana et al. (20) showed that poor sleep quality was linked to greater anxiety and depression. The objective of this study is therefore to examine the relationship between sleep quality and other non-motor symptoms at baseline, and to determine if poor sleep quality influences the evolution of other non-motor symptoms over time in an early PD cohort.

## Methods

The Early Parkinson's Disease Longitudinal Singapore (PALS) study is a prospective cohort study in early PD. All subjects were recruited from movement disorder clinics at the National Neuroscience Institute, Singapore. Data was accessed on 1st August 2019 and included 109 subjects who had completed one-year follow-up assessments. Inclusion criteria were (1) Age between 21 to 90 years old; (2) Fulfill the National Institute of Neurological Disorders and Stroke (NINDS) diagnostic criteria for PD; (3) Recruited within 1 year from PD diagnosis; (4) Cardinal motor symptoms of PD (rest tremor, rigidity or bradykinesia) present for <2 years before diagnosis. Exclusion criteria included a history of stroke, active malignancy, significant orthopedic abnormalities affecting movement, end-organ failure, and significant neurological or psychiatric comorbidity.

Demographic information was collected, with clinical assessments performed annually. Motor assessments included the neurological examination, Movement Disorders Society Unified Parkinson's Disease Rating Scale (MDS-UPDRS) (21) and Hoehn & Yahr (H&Y) Scale (22). Levodopa Equivalent Dosage (LEDD) was calculated using established methods (23).

Sleep quality was assessed using the Pittsburgh Sleep Quality Index (PSQI), a self-rated questionnaire evaluating sleep quality that has been validated for use in PD (24). Patients were stratified into two groups based on baseline PSQI scores (25): scores ≤5 indicating good sleepers, and >5 indicating poor sleepers. Excessive daytime sleepiness (EDS) was also evaluated using the Epworth Sleepiness Scale (ESS), with established cut-off scores of > 10 considered as EDS (26). Fatigue was assessed with the Fatigue Severity Scale (FSS), with scores ≥4 indicating significant fatigue (27). Other non-motor assessments performed included the Non-Motor Symptom Scale (NMSS) (28), Geriatric Depression Scale (GDS) (29), Hospital Anxiety and Depression Scale (HADS) (30) and Apathy Scale (AS) (31).

Global cognition was assessed using the Mini Mental State Examination (MMSE) (32) and the Montreal Cognitive Assessment (MoCA) (33). PD-mild cognitive impairment (PD-MCI) was defined according to the Movement Disorders Society Level II criteria, which requires a score of <1.5 standard deviations on at least 2 neuropsychological tests, using norms corrected for gender, age, education and cultural differences (34). The tests performed included:

1. Memory: Alzheimer's Disease Assessment Scale-Cognitive Subscale (ADAS-Cog) Immediate and Delayed recall (35); Rey-Osterreith Complex Figure (ROCF) Copy and Recall (36).
2. Visuospatial Function: Repeatable Battery for the Assessment of Neuropsychological Status (RBANS) Judgement of Line Orientation (37); Clock Drawing Task (CLOX-2) (38); Alzheimer's Disease Assessment Scale-Cognitive Subscale (ADAS-Cog) Constructional Praxis (35).
3. Attention and Working Memory: Wechsler Adult Intelligence Scale 4^th^ Edition (WAIS-IV) Digit Span; Wechsler Memory Scale 4^th^ Edition (WMS-IV) Symbol Span (39).
4. Language: Boston Naming Test (BNT) (40); Wechsler Adult Intelligence Scale 4^th^ Edition (WAIS-IV) Similarities (39).
5. Executive Functioning: Frontal Assessment Battery (FAB) (41); Fruit Fluency test (42).

Baseline demographic and clinical data was compared using independent samples *t*-test or Mann-Whitney *U* test for continuous variables (depending on whether the normality assumption was tenable), and Chi-square test was used for categorical variables. To compare differences in the clinical assessments between poor and good sleepers at baseline, a multivariable linear regression model was constructed, adjusted for age, gender, disease duration- defined as time since formal diagnosis of PD by a qualified neurologist, years of education, LEDD, baseline MDS-UPDRS Part III, and MoCA scores. Multi-collinearity among covariates was assessed using the variance inflation factor *via* multivariable linear regression models.

The longitudinal effect of sleep quality on the progression of non-motor symptoms was investigated with multivariable regression models, and least-square means (95% CI) of the outcome variable were reported for good and poor sleepers. All longitudinal analysis was adjusted for age, gender, disease duration, years of education, LEDD, baseline MDS-UPDRS Part III, MoCA scores, as well as the baseline scores for the respective non-motor test being evaluated. Statistical significance was set at *p* < 0.05.

All analysis was performed using SAS software version 9.4 for Windows (Cary, NC: SAS Institute Inc.). The Singapore Health Services Centralized Institutional Review Board provided ethics approval, and written informed consent was obtained from all participants.

## Results

### Cohort demographics

109 subjects (67 male; 42 females) were included in the study, with a mean age of 66.0 ± 9.1 years. The mean disease duration was 6.0 ± 3.5 months and the mean MDS-UPDRS Part III score was 20.3 ± 9.7. Using baseline PQSI scores, 59 poor sleepers and 50 good sleepers were identified: there were no significant differences in baseline demographics, LEDD, MDS-UPDRS Part III scores, H&Y stage, global cognitive performance and the prevalence of PD-MCI between the two groups (*p* > 0.05). Details are presented in Table 1.

**Table 1:** Demographics and baseline clinical features in good and poor sleepers.

	**Cohort**	**Good sleepers**	**Poor sleepers**	*p*-value^*^
	**(*n* = 109)**	**(*n* = 50)**	**(*n* = 59)**	
Age at baseline visit, year	66.0 ± 9.1^†^	65.7 ± 10.1	66.2 ± 8.1	0.80
Male gender, *n* (%)	67 (61.5)	32 (64.0)	35 (59.3)	0.38
Education, year	11.3 ± 4.2	11.9 ± 3.8	10.8 ± 4.3	0.11
Disease duration, months	6.0 ± 3.5	6.4 ± 3.8	5.7 ± 3.1	0.97
LEDD, mg/d	227.3 ± 178.7	202.7 ± 182.2	233.2 ± 175.1	0.19
MDS-UPDRS Part III	20.3 ± 9.7	19.7 ± 9.1	20.1 ± 9.8	0.60
H & Y stage	1.9 ± 0.4	1.8 ± 0.4	1.8 ± 0.4	0.52
MoCA	26.1 ± 2.7	26.3 ± 2.5	25.9 ±2.9	0.48

* Two independent samples t-test or Mann-Whitney U test for continuous variables (depending on normality assumption), and Chi-square test for categorical variables.

† Continuous variables reported as mean ± standard deviation; categorical variables reported as frequency (%).

LEDD, Levodopa equivalent daily dosage; MDS-UPDRS, Movement Disorders Society Unified Parkinson's Disease Rating Scale; H & Y Stage, Hoehn and Yahr stage; MoCA, Montreal Cognitive Assessment; PD-MCI, Parkinson's Disease mild cognitive impairment.

### Comparison of baseline non-motor symptoms

In the multivariable analysis adjusted for age, gender, disease duration, LEDD, UPDRS Part III scores, years of education and baseline MoCA scores: NMSS score was 23.0 (18.3, 27.8) for poor sleepers and 13.7 (8.3, 19.1) for good sleepers (*p* = 0.011) and the HADS anxiety score was 2.99 (2.26, 3.73) for poor sleepers and 1.59 (0.75, 2.42) for good sleepers (*p* = 0.013). There were no differences in baseline GDS, FSS, AS and ESS scores between the two groups (*p* > 0.05) (Table 2). As for cognition, there were no significant differences in test scores across all domains and tests (Table 3), except for the WAIS-IV Backward Digit Span with poor sleepers scoring 7.72 (7.19, 8.25) and good sleepers scoring 8.57 (7.97, 9.16), (*p* = 0.038).

**Table 2:** Baseline differences in non-motor symptoms between good and poor sleepers.

**Non-motor tests**	**Good sleepers**	**Poor sleepers**	*p*-value^*^
	**(*n* = 50)**	**(*n* = 59)**	
NMSS total	14.31^†^(9.01, 19.61)	23.64 (18.92, 28.36)	**0.011**
NMSS D1 (cardiovascular)	0.27 (−0.18, 0.73)	0.85 (0.44, 1.26)	0.070
NMSS D2 (sleep/fatigue)	0.78 (−0.58, 2.15)	4.23 (3.01, 5.44)	**<0.001**
NMSS D3 (Mood/cognition)	0.52 (−0.33, 1.37)	1.60 (0.85, 2.36)	0.064
NMSS D4 (Perceptual problems)	0.70 (−0.17, 1.57)	0.83 (0.06, 1.61)	0.826
NMSS D5 (Attention/memory)	1.27 (0.15, 2.40)	1.97 (0.97, 2.97)	0.366
NMSS D6 (Gastrointestinal)	1.89 (0.76, 3.02)	2.43 (1.42, 3.43)	0.491
NMSS D7 (Urinary)	4.80 (2.46, 7.14)	6.24 (4.16, 8.33)	0.366
NMSS D8 (Sexual)	1.52 (0.24, 2.81)	2.11 (0.97, 3.25)	0.503
NMSS D9 (Miscellaneous)	2.54 (1.09, 3.99)	3.38 (2.09, 4.68)	0.397
GDS	1.25 (0.82, 1.68)	1.79 (1.40, 2.17)	0.068
HADS anxiety	1.50 (0.68, 2.32)	2.91 (2.18, 3.63)	**0.013**
FSS	23.16 (19.11, 27.22)	28.51 (24.91, 32.12)	0.055
AS	7.38 (5.64, 9.11)	7.80 (6.26, 9.11)	0.720
ESS	5.18 (4.22, 6.14)	5.25 (4.40, 6.10)	0.914

* Adjusted for age, gender, disease duration, LEDD, Levodopa equivalent daily dosage, MDS-UPDRS, Movement Disorders Society Unified Parkinson's Disease Rating Scale, Years of education, MoCA, Montreal cognitive assessment.

† Least-square means (95% confidence interval) using multivariable linear regression analysis.

NMSS, Non-Motor Symptom Scale; GDS, Geriatric Depression Scale; HADS, Hospital Anxiety and Depression Scale; FSS, Fatigue Severity Scale; AS, Apathy Scale; ESS, Epworth Sleepiness Scale. The bold values indicate statistical significance.

**Table 3:** Baseline differences in cognition between poor and good sleepers.

**Cognitive tests**	**Good sleepers**	**Poor sleepers**	*p*-value^*^
	***n*** **=** **50**	***n*** **=** **59**	
PD-MCI, n (%)	20 (40.0)	21 (35.6)	0.421
Global:			
MOCA	26.41 (25.68, 27.14)	26.03 (25.38, 26.68)	0.444
Attention:			
WAIS-IV forward digit span	10.96 (10.25, 11.67)	10.27 (9.64, 10.90)	0.155
WAIS-IV backward digit span	8.57 (7.97, 9.16)	7.72 (7.19, 8.25)	**0.038**
WMS-IV symbol span	18.16 (15.65, 20.68)	16.11 (13.86, 18.35)	0.232
Executive function:			
Fruit fluency	13.98 (13.13, 14.84)	14.66 (13.89, 15.42)	0.251
FAB	16.41 (15.96, 16.86)	16.51 (16.11, 16.91)	0.749
Language:			
BNT total score	25.76 (24.87, 26.64)	26.42 (25.63, 27.20)	0.274
WAIS-IV similarities	20.72 (19.09, 22.34)	21.24 (19.79, 22.69)	0.637
Memory:			
ROCF copy	32.34 (31.18, 33.30)	32.15 (31.20, 33.09)	0.897
ROCF delayed recall	15.23 (13.11, 17.34)	15.14 (13.26, 17.02)	0.955
ADAS-Cog immediate recall	3.15 (2.78, 3.51)	2.87 (2.54, 3.19)	0.264
ADAS-Cog delayed recall	2.93 (2.41, 3.45)	2.62 (2.15, 3.08)	0.381
Visuospatial:			
RBANS line judgement	15.68 (14.82, 16.55)	15.39 (14.62, 16.16)	0.617
ADAS-Cog constructional praxis	3.63 (3.46, 3.79)	3.60 (3.46, 3.75)	0.840
CLOX-2	14.71 (14.52, 14.90)	14.62 (14.45, 14.79)	0.511

* Adjusted for age, gender, disease duration, LEDD, Levodopa equivalent daily dosage, MDS-UPDRS, Movement Disorders Society Unified Parkinson's Disease Rating Scale, Years of education.

Least-square means (95% confidence interval) using multivariable linear regression analysis.

PD-MCI, Parkinson Disease- Mild Cognitive Impairment; MOCA, Montreal Cognitive Assessment; WAIS-IV, Weschler Adult Intelligence Scale 4^th^ Edition; FAB, Frontal Assessment Battery; ROCF, Rey-Osterrieth Complex Figure; ADAS-Cog, Alzheimer's Disease Assessment Scale- Cognitive; RBANS, Repeatable Battery for Assessment of Neuropsychological Status; CLOX-2, Clock Drawing Test.

The bold values indicate statistical significance.

### Change in non-motor symptoms over time

Poor sleep quality at baseline was associated with greater fatigue and depression at 1 year follow-up. After 1 year, FSS scores increased by 3.60 points in poor sleepers but decreased by 2.93 points in good sleepers when compared to baseline values (*p* = 0.007); GDS scores increased by 0.42 points in poor sleepers but decreased by 0.70 points in good sleepers (*p* = 0.006) (Table 4). There were no significant differences in the change in cognitive performance, anxiety, apathy and EDS after one year between good and poor sleepers (all *p* >0.05) (Table 5).

**Table 4:** Multivariable analysis of the effect of baseline sleep quality on the change in clinical test scores on 1-year follow-up.

**Non-motor test**	**Good sleepers**	**Poor sleepers**	*p*-value^*^
	**(*n* = 50)**	**(*n* = 59)**	
MoCA	−0.32^†^(−0.89, 0.25)	−0.46 (−0.96, 0.04)	0.718
NMSS total	3.54 (−1.96, 9.05)	4.01 (−0.88, 8.89)	0.903
NMSS D1 (cardiovascular)	0.73 (−0.19, 1.65)	0.47 (−0.35, 1.29)	0.677
NMSS D2 (sleep/fatigue)	0.06 (−1.41, 1.53)	1.90 (0.60, 3.20)	0.077
NMSS D3 (Mood/cognition)	1.09 (−0.58, 2.75)	1.97 (0.49, 3.45)	0.442
NMSS D4 (Perceptual problems)	−0.39 (−0.90, 0.13)	−0.40 (−0.86, 0.06)	0.958
NMSS D5 (Attention/memory)	−0.38 (−1.11, 0.36)	−0.46 (−1.12, 0.19)	0.862
NMSS D6 (Gastrointestinal)	0.94 (−0.04, 1.91)	0.85 (−0.02, 1.71)	0.892
NMSS D7 (Urinary)	−0.57 (−2.65, 1.51)	−0.02 (−1.88, 1.83)	0.700
NMSS D8 (Sexual)	−0.84 (−1.87, 0.19)	−0.64 (−1.55, 0.28)	0.778
NMSS D9 (Miscellaneous)	1.36 (−0.49, 3.21)	1.58 (−0.07, 3.23)	0.864
GDS	−0.63 (−1.22, −0.05)	0.49 (−0.03, 1.00)	**0.006**
HADS anxiety	0.22 (−0.43, 0.86)	0.40 (−0.17, 0.97)	0.672
FSS	−3.08 (−6.55, 0.39)	3.45 (0.37, 6.53)	**0.007**
AS	−0.43 (−2.09, 1.24)	1.48 (−0.01, 2.96)	0.096
ESS	0.11 (−0.99, 1.20)	0.09 (−0.90, 1.07)	0.977

* Adjusted for age, gender, disease duration, LEDD, Levodopa equivalent daily dosage, MDS-UPDRS, Movement Disorders Society Unified Parkinson's Disease Rating Scale, Years of education, MoCA, Montreal cognitive assessment and baseline outcome scale.

† Least-square means (95% confidence interval) using multivariable linear regression analysis.

MoCA, Montreal Cognitive Assessment; NMSS, Non-Motor Symptom Scale; GDS, Geriatric Depression Scale; HADS, Hospital Anxiety and Depression Scale; FSS, Fatigue Severity Scale; AS, Apathy Scale; ESS, Epworth Sleepiness Scale.

The bold values indicate statistical significance.

**Table 5:** Multivariable analysis of the effect of baseline sleep quality on the change in cognitive test scores on 1-year follow-up.

**Cognitive tests**	**Good sleepers**	**Poor sleepers**	*p*-value^*^
	***n* = 50**	***n* = 59**	
PD-MCI (%)	15 (30.0)	20 (33.8)	0.838
Attention:			
WAIS-IV forward digit span	0.39^†^(−0.20, 0.99)	0.06 (−0.47, 0.59)	0.413
WAIS-IV backward digit span	0.12 (−0.46, 0.69)	0.18 (−0.34, 0.69)	0.871
WMS-IV symbol span	1.10 (−1.00, 3.20)	0.65 (−1.22, 2.52)	0.750
Executive function:			
Fruit fluency	0.11 (−0.76, 0.99)	−0.18 (−0.95, 0.60)	0.629
FAB	−0.15 (−0.67, 0.37)	0.14 (−0.33, 0.60)	0.423
Language:			
BNT total score	0.20 (−0.47, 0.87)	0.21 (−0.38, 0.81)	0.982
WAIS-IV similarities	0.30 (−0.78, 1.39)	0.20 (−0.77, 1.17)	0.886
Memory:			
ROCF copy	−0.10 (−1.21, 1.02)	−0.75 (−1.74, 0.25)	0.393
ROCF delayed recall	2.51 (0.85, 4.17)	1.05 (−0.42, 2.53)	0.200
ADAS-Cog immediate recall	−0.03 (−0.36, 0.30)	−0.06 (−0.35, 0.23)	0.892
ADAS-Cog delayed recall	−0.53 (−1.02, −0.04)	−0.10 (−0.53, 0.35)	0.194
Visuospatial:			
RBANS line judgement	0.23 (−0.50, 0.96)	0.27 (−0.38, 0.92)	0.934
ADAS-Cog constructional praxis	0.03 (−0.12, −0.19)	−0.01 (−0.15, 0.13)	0.677
CLOX-2	−0.04 (−0.25, 0.17)	0.01 (−0.17, 0.20)	0.719

* Adjusted for age, gender, disease duration, LEDD, Levodopa equivalent daily dosage, MDS-UPDRS, Movement Disorders Society Unified Parkinson's Disease Rating Scale, Years of education and baseline outcome scale.

† Least-square means (95% confidence interval) using multivariable linear regression analysis.

PD-MCI, Parkinson Disease- Mild Cognitive Impairment; MOCA, Montreal Cognitive Assessment; WAIS-IV, Weschler Adult Intelligence Scale 4^th^ Edition; FAB, Frontal Assessment Battery; ROCF, Rey-Osterrieth Complex Figure; ADAS-Cog, Alzheimer's Disease Assessment Scale- Cognitive; RBANS, Repeatable Battery for Assessment of Neuropsychological Status; CLOX-2, Clock Drawing Test.

## Discussion

In this longitudinal study of sleep quality in a well-characterized early PD cohort with cognitive tests performed according to MDS Level II criteria for PD-MCI, we show that: (1) Poor baseline sleep quality was associated with increased anxiety at baseline; (2) Poor baseline sleep quality was associated with greater fatigue and depression after 1 year. (3) Poor baseline sleep quality was not associated with differences in global and domain cognition both at baseline, and was also not associated with worsening cognitive function after 1 year in both global and domain measures.

At baseline, poor sleepers displayed greater anxiety, but this did not worsen 1 year later as compared to good sleepers. A cross-sectional study in non-early PD showed that anxiety was a significant predictor of poorer sleep quality (10), while in early PD, a bidirectional longitudinal study by Rutten et al. showed that whilst anxiety is a predictor for insomnia at a follow-up duration of 6 months, the converse is not true (43). Our study agrees with the previous findings in early PD, with a baseline relationship shown between poor sleep quality and anxiety, and that poor sleep quality was not a predictor of worse anxiety at one-year follow-up.

Our study shows that early PD patients with poor sleep quality do not have higher fatigue scores at baseline, but rather, have greater worsening of their fatigue at follow-up as compared to patients with good sleep quality. Studies of sleep quality and fatigue in early PD have been conflicting: one study found an association between poor sleep quality and fatigue (44), but another study did not (45).

Fatigue was still reported in one-fifth of good sleepers, and no baseline difference in fatigue scores were seen between poor and good sleepers, suggesting that sleep quality is not the sole determinant of fatigue in early PD. Fatigue remains poorly understood, and is thought to be a result of serotonergic dysfunction in the basal ganglia and limbic system (13). Decreased striatal and limbic serotonergic activity has been found in non-PD subjects with chronic fatigue syndrome (46) and also in PD patients with fatigue (47). In the latter study, no differences in dopaminergic activity were detected between fatigued and non-fatigued PD patients, which suggests that serotonin is likely to be the predominant neurotransmitter responsible for fatigue in PD. Other recent longitudinal studies on fatigue in early PD have shown conflicting relationships between dopamine and fatigue. Ongre et al. (48) showed that in *de novo* PD patients, lower levels of fatigue were seen in patients using dopamine agonists than levodopa, and Ou et al. (49) showed higher fatigue prevalence in patients with higher LEDD at the 2 and 3-year mark of follow up. In our study, the effect of dopaminergic drugs was corrected for using LEDD in the statistical model, and an association between sleep quality and fatigue was shown. It is well established that serotonergic dysfunction contributing to the development of sleep disorders (50). It is thus possible that poor sleep quality and fatigue coexist in PD due to underlying serotonergic dysfunction, rather than fatigue resulting simply as a consequence of poor sleep quality. Further research into its pathophysiology may shed light on whether sleep and fatigue share similar neuropathological processes, or if sleep quality is the greatest determinant of fatigue.

Likewise, serotonergic dysfunction is well known to be implicated in the pathogenesis of psychiatric depression and anxiety disorders, with pharmacologic management largely targeted at increasing serotonergic activity in the brain. With regards to depression and anxiety in PD, serotonin is also thought to be a major factor in their occurrence (51). Neuroimaging studies in early PD patients have shown that alterations in serotonergic function may even be present in the early stages of disease, and that these alterations are more evident in patients who also report affective symptoms such as anxiety and depression (52). Our study found that poor sleep quality was associated with increased anxiety but not increased depressive symptoms at baseline, and that depressive symptoms worsened in poor sleepers compared to good sleepers after 1-year follow-up. Our study supports the notion that serotonin is involved in the pathogenesis of sleep disorders, fatigue, depression and anxiety. We show a clear association between poor sleep quality and worsening fatigue and depression over time in an early PD cohort, as well as higher baseline anxiety. In patients presenting with this constellation of non-motor symptoms, the role of therapies directed at correcting serotonergic dysfunction will need to be explored further to determine if they are effective in improving the quality of life of our patients.

Associations between sleep quality and cognition in PD are conflicting. In non-early PD cohorts, some studies have linked poor sleep with poorer cognitive function (10, 19), particularly in the domain of attention and executive function (18). However, other studies in moderate to advanced PD, failed to identify this association (53, 54). In our study in early PD, there was no association between sleep quality and cognitive function at baseline or one-year follow up, even when using the more rigorous MDS Level II criteria for mild cognitive impairment. Given that cognitive decline develops later in disease than sleep disturbances, it is possible that such an association is not yet apparent (55), and further longitudinal follow up is necessary.

There were some limitations in our study. We had utilized self-reported sleep questionnaires rather than the gold standard polysomnography. Nonetheless, the PSQI has been validated in PD and reflects typical clinical reports of sleep disturbances encountered by clinicians. In addition, the data analyzed was limited to follow-up visits at 1 year. Further research is also being carried out on how the change in sleep quality over time affects the progression of the various non-motor symptoms, as sleep quality may change with progression of disease. It is also possible that the effects of sleep quality on other aspects of disease may be more evident over time, and the various non-motor manifestations of disease are not yet apparent within such a short duration. Analysis of patients at across a longer duration may yield more findings.

## Conclusion

In conclusion, we show that poor sleep quality is associated with greater anxiety at baseline, as well as the progression of fatigue and depression over time in early PD patients, reinforcing the hypothesis of serotonergic dysfunction in the basal ganglia and limbic system in the pathogenesis of these non-motor symptoms of PD. Even though evidence seems to point toward serotonin as the chief neurotransmitter involved in the genesis of these symptoms, their exact pathophysiology is poorly understood, particularly for fatigue. Further exploration of the mechanisms by which these constellations of symptoms develop may elucidate pertinent information which may aid in treatment, with a possible emphasis on correcting serotonergic dysfunction.

## Data availability statement

The raw data supporting the conclusions of this article will be made available by the authors, without undue reservation.

## Ethics statement

The studies involving human participants were reviewed and approved by Singapore Health Services Centralized Institutional Review Board. The patients/participants provided their written informed consent to participate in this study.

## Author contributions

MK drafted the manuscript and assisted with conception and design of the study and with data processing. CC assisted with data processing and conception and design of the study. SY-EN, NC, RC, XC, DH, SXN, KT, WA, E-KT, LT, and ZX were involved in participant recruitment to the PALS study and gathering data from participants. SS oversaw statistical design and analysis of the raw data. LT oversaw the overall PALS study. ZX contributed greatly to revisions to the manuscript and conception and design of the study. All joint senior authors read and approved the submitted version.

## Funding

This study was supported by the Singapore Ministry of Health's National Medical Research Council under its Translational and Clinical Research Flagship Programme (NMRC/TCR/013-NNI/2014) and Open Fund Large Collaborative Grant (MOH-OFLCG18May-000s2).

## Conflict of interest

The authors declare that the research was conducted in the absence of any commercial or financial relationships that could be construed as a potential conflict of interest.

## Publisher's note

All claims expressed in this article are solely those of the authors and do not necessarily represent those of their affiliated organizations, or those of the publisher, the editors and the reviewers. Any product that may be evaluated in this article, or claim that may be made by its manufacturer, is not guaranteed or endorsed by the publisher.
